# An automated stochastic approach to the identification of the protein specificity determinants and functional subfamilies

**DOI:** 10.1186/1748-7188-5-29

**Published:** 2010-07-15

**Authors:** Pavel V Mazin, Mikhail S Gelfand, Andrey A Mironov, Aleksandra B Rakhmaninova, Anatoly R Rubinov, Robert B Russell, Olga V Kalinina

**Affiliations:** 1 Department of Bioengineering and Bioinformatics, Moscow State University, Moscow, Russia; 2 Cellnetworks, University of Heidelberg, Heidelberg, Germany; 3 Institute for Information Transmission Problems RAS, Moscow, Russia

## Abstract

**Background:**

Recent progress in sequencing and 3 D structure determination techniques stimulated development of approaches aimed at more precise annotation of proteins, that is, prediction of exact specificity to a ligand or, more broadly, to a binding partner of any kind.

**Results:**

We present a method, SDPclust, for identification of protein functional subfamilies coupled with prediction of specificity-determining positions (SDPs). SDPclust predicts specificity in a phylogeny-independent stochastic manner, which allows for the correct identification of the specificity for proteins that are separated on a phylogenetic tree, but still bind the same ligand. SDPclust is implemented as a Web-server http://bioinf.fbb.msu.ru/SDPfoxWeb/ and a stand-alone Java application available from the website.

**Conclusions:**

SDPclust performs a simultaneous identification of specificity determinants and specificity groups in a statistically robust and phylogeny-independent manner.

## Background

The current explosion of data on protein sequences and structures lead to the emergence of techniques that go beyond standard annotation approaches, i.e. annotation by close homolog and homology-based family identification. These approaches usually start with a set of related sequences and perform a detailed analysis of each alignment position [1-15]. One of problems that such analysis can tackle is analysis of protein specificity. Let us assume that a protein family has undergone an ancient duplication that resulted in proteins that are related but perform different functions in the same organism. It is natural to assume that this functional divergence is mediated by mutation of certain amino acid positions. We call these positions specificity determinants, and this study is focused on their identification. We assume that specificity determinants, after mutation that allow for a new (sub-)function, should be under strong negative selection to let this newly asserted function to persist. This results is a very specific conservation pattern of the position in a multiple sequence alignment of the protein family: it is conserved among proteins that perform exactly same function and differ between different functional sub-groups. In this study, such positions are called SDPs (Specificity-Determining Positions). Another facet of the same problem is identification of proteins that have a certain specificity, i.e. refined functional annotation.

Most of techniques dealing with the stated problem reduce the problem of specificity prediction to the identification of alignment positions that may be important for protein specificity. They require the input set of sequences to be divided into groups of proteins having the same specificity (specificity groups) [1,3,4,6,9-15]. A common feature of these methods is that they measure the correlation between the distribution of amino acids in each position of a multiple sequence alignment (MSA) and the pre-defined groups. Those positions that show relatively high correlation are assumed to be important for differences in specificity between groups. Additionally, SDPpred [6] allows for a subsequent prediction of specificity for proteins, whose specificity has not been known a priori.

Some methods do not need prior information on protein specificity [2,5,7,12]. They start with an automated division of the MSA into possible specificity groups. A common feature of these methods is that they assign same specificity only to monophyletic clades of the proteins' phylogenetic tree. This imposes a significant restriction if the distribution of specificities within the protein family does not agree with the phylogeny. This can happen either as a consequence of convergent evolution, or if the phylogeny is not well resolved.

In this paper we address these weaknesses. We present a method, SDPclust, that simultaneously identifies SDPs and divides the alignment into groups of proteins that have the same specificity in a phylogeny-independent manner. Other phylogeny-independent methods to identify specificity-determining sites have been developed by Marttinen and co-workers [16] and Reva and co-workers [17]. We report the benchmarking of the presented method below.

## Methods

### Algorithm

Previously, we introduced the concept of specificity-determining positions (SDPs) [6,18]. Briefly, we say that a position of a multiple sequence alignment (MSA) is an SDP, if amino acids in the corresponding MSA column are conserved within pre-defined groups of proteins with the same specificity (specificity groups) and differ between such groups. We assume that positions with such conservation pattern account for differences in the specificity between proteins from different specificity groups.

One can easily note that the definition of SDPs relies on the definition of specificity groups in a protein family. This significantly constrains the applicability of previously developed methods. On the other hand, we previously showed that the identification of specificity groups can be done using SDPs [6,18]. SDPclust is a novel method that identifies SDPs in the absence of prior knowledge of specificity groups and simultaneously predicts these groups. At that, SDPclust does not predict the protein specificity *ab initio*, it merely says that proteins have coinciding or different specificity.

**Figure 1:**
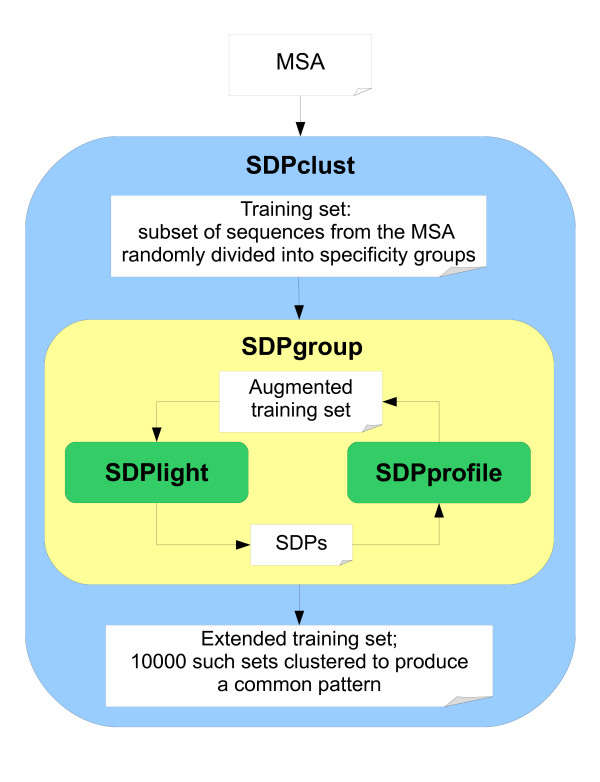
**Blocks and connections in the SDPclust algorithm**.

SDPclust consists of several components, which are connected as shown in Figure 1. SDPlight is a fast procedure to identify SDPs in a MSA that is divided into specificity groups. The idea of SDPlight is the same as in a previously reported method SDPpred [6], namely, it uses the mutual information to measure how close is the distribution of amino acids in a given MSA position *p *to the distribution of proteins into specificity groups:

(1) $$MI_{p}=\sum_{\begin{array}{l} \alpha \in \\ \text{all amino acids} \end{array}}\sum_{\begin{array}{l} i\in \\ \text{all}\\ \text{specificity groups} \end{array}}f_{p}(\alpha ,i)\log\frac{f_{p}(\alpha ,i)}{f_{p}(\alpha )f(i)},$$

where *f*_*p*_(*α*, *i*) is the frequency of amino acid α in group *i *in position *p*, *f*_*p*_(*α*) is the frequency of amino acid *a *in position *p *in the whole alignment, *f (i) *is the fraction of proteins in group *i*.

The main new feature of SDPlight that makes it much faster than SDPpred is the way the correction for the background distribution of the mutual information is performed. Instead of using shuffling, which is computationally inefficient, we pre-calculate the mean and the variance for any pattern of amino acids in an arbitrary column using an approximation described below. Let us assume that a MSA consists of proteins falling into *k *specificity groups, and, in a given position, amino acid *α *appears in each group *i*_*αj *_times (*j *= 1,...,*k*). Then (1) can be rewritten as

(2) $$MI_{p}=\frac{1}{N}\sum_{\alpha =1}^{20}\sum_{j=1}^{k}MI(\alpha ,j),$$

where

(3) $$MI(\alpha ,j)=i_{\alpha j}\log\left(\frac{i_{\alpha j}n}{i_{\alpha }n_{j}}\right),$$

where *n*_*j *_is the size of group *j*, Σ_*j *= 1,...,*k *_*n*_*j *_= *N*, *N *is the total number of sequences in the MSA.

The exact formulae for the expectation value and the variance of *MI*_*p *_are:

(4) $$M(MI_{p})=\frac{1}{N}\sum_{\alpha =1}^{20}\sum_{j=1}^{k}\sum_{\left\{i_{\alpha j}\right\}}p(\{i_{\alpha j}\})MI(\alpha ,j),$$

(5) $$\begin{array}{l} D(MI_{p})=\frac{1}{N^{2}}(\sum_{\alpha =1}^{20}\sum_{j=1}^{k}D(MI(\alpha ,j))\\ +2\sum_{\begin{array}{l} \alpha_{1},\alpha_{2}=1\\ \alpha_{1}>\alpha_{2} \end{array}}^{20}\sum_{{}^{\begin{array}{l} j_{{}_{1}},j_{{}_{2}}=1\\ j_{{}_{1}}>j_{{}_{2}} \end{array}}}^{k}cov(MI(\alpha_{1},j_{1}),MI(\alpha_{2},j_{2}))). \end{array}$$

At this point we make the approximation that different amino acids are distributed independently in the groups. This leads to several simplifications: groups become equivalent, hence *n*_*j*_/*n *= 1/*k*. Since all groups become equivalent, instead of taking a sum over all groups, we can multiply by *k*. The distribution {*i*_*αj*_} of amino acids in *k *groups can be approximated by a multinomial distribution. Since the distributions for different amino acids are independent, we can rewrite formula (4) as $M(MI_{p})=\frac{1}{N}\cdot \sum_{\alpha =1}^{20}M\left(MI_{i_{\alpha ,}k}\right),$, probability to observe a given pattern of amino acid *a *is binomial and can be approximated as:

(6) $$p\left(\left\{i_{\alpha j}\right\}\right)={\tilde{p}}_{i_{\alpha ,}k}\left(i\right)=\left(\begin{array}{l} i_{\alpha }\\ i \end{array}\right){\left(\frac{1}{k}\right)}^{i}{\left(1-\frac{1}{k}\right)}^{i_{\alpha }-i}.$$

So the approximation for the expectation value of *MI*_*p *_is:

(7) $$\begin{array}{l} M(MI_{p})=\frac{1}{N}\cdot \sum_{\alpha =1}^{20}M\left(MI_{i_{\alpha ,}k}\right)=\\ =\frac{k}{N}\sum_{\alpha =1}^{20}\sum_{i=1}^{i_{\alpha }}\frac{i_{\alpha }!}{i!\left(i_{\alpha }-i\right)!}{\left(\frac{1}{k}\right)}^{i}{\left(1-\frac{1}{k}\right)}^{i_{\alpha }-i}i\log\left(\frac{ik}{i_{\alpha }}\right). \end{array}$$

Since the distributions of amino acids are independent, all covariances between two amino acids in formula (5) equal 0, and all covariances between groups are equal to each other (so we can effectively multiply by *k*^2 ^- *k*):

(8) $$\begin{array}{l} D(MI_{p})=\frac{1}{N^{2}}\sum_{\alpha =1}^{20}D(MI_{i_{\alpha },k})=\\ \frac{1}{N^{2}}\sum_{\alpha =1}^{20}D\;\left(\sum_{i=1}^{i_{\alpha }}\frac{i_{\alpha }!}{i!(i_{\alpha }-i)!}{\left(\frac{1}{k}\right)}^{i}{\left(1-\frac{1}{k}\right)}^{i_{\alpha }-i}i\log\left(\frac{ik}{i_{\alpha }}\right)\right)=\\ =\frac{k}{N^{2}}\sum_{\alpha =1}^{20}\sum_{i=1}^{i_{\alpha }}\frac{i_{\alpha }!}{i!(i_{\alpha }-i)!}{\left(\frac{1}{k}\right)}^{i}{\left(1-\frac{1}{k}\right)}^{i_{\alpha }-i}{\left(i\log\left(\frac{ik}{i_{\alpha }}\right)\right)}^{2}+\\ +(k^{2}-k)\sum_{\alpha =1}^{20}\sum_{i=1}^{i_{\alpha }}\sum_{i=2}^{i_{\alpha }-i_{1}}\frac{i_{\alpha }!}{i_{1}!\;i_{2}!(i_{\alpha }-i_{1}-i_{2})!}{\left(\frac{1}{k}\right)}^{i_{1}+i_{2}}.\\ .{\left(1-\frac{2}{k}\right)}^{i_{\alpha }-i_{1}-i_{2}}i_{1}\log\left(\frac{i_{1}k}{i_{\alpha }}\right)i_{2}\log\left(\frac{i_{2}k}{i_{\alpha }}\right)-M^{2}\left(MI_{i_{\alpha },k}\right) \end{array}$$

The values of *M*($MI_{i_{\alpha },k}$) and *D*($MI_{i_{\alpha },k}$) are pre-calculated and tabulated, and requiring time *O*(*i*_*α*_) and *O*(*i*_*α*_^2^), respectively. We pre-calculate these values for *k *= 2,...,200; *i*_*α *_= 1,...,500 and store them. Then one run of the method involves only summing corresponding pre-calculated values for all 20 amino acids, and for a given alignment of ~100 sequences of length ~400 aa it takes approximately 50 ms (AMD Athlon™ 64 Processor 3800+).

Having pre-calculated values of *M*(*MI*) and *D*(*MI*), and given a MSA, we can calculate Z-scores for each position and the probability to obtain *k *highest-scoring positions analogously to SDPpred [6]. Finally, we select the least probable, given a random model, set of positions and call them SDPs, for these are the positions that correlate best with grouping of sequences by specificity.

The second component of the method is SDPprofile, which, analogously to SDPpred, computes positional weight matrices (PWMs) for each specificity group based only on the predicted SDPs and ignoring the rest of the alignment. Then, for a protein of unknown specificity, it is possible to assign it to one of the specificity groups by the highest PWM score. This allows us to augment the initial specificity groups with additional sequences. A virtual group was introduced to account for sequences that cannot be grouped into existing groups. It contains all sequences of the alignment and is ignored during the prediction of SDPs. Any sequence that has significantly higher score for one of the constructed PWMs is assigned to that group, whereas sequences with low scores or without pronounced preference of one PWM are assigned to the virtual group.

Whereas the components described above reproduce to some extent the previously reported method, the following components are new and allow for considering protein families that lack prior information on specificity.

SDPgroup is an iterative procedure to augment a pre-defined training set of specificity groups with new sequences from the MSA, and SDPtree is a wrapping procedure that multiply picks a random training set for SDPgroup and constructs the best clustering pattern.

The iterative steps of SDPgroup are the following. We start from a given training set of specificity groups and identify SDPs using the SDPlight procedure. Then we consider each sequence of the MSA as a sequence of unknown specificity, and use SDPprofile to reassign it to one of the specificity groups. After this step, most of sequences would probably stay in same groups, but specificity assignment of some sequences may change, and other sequences of previously unknown specificity may get assigned to one of the groups. The reassignment of all sequences constitutes one iterative step. Then we recalculate SDPs. If the grouping of sequences does not change after the current iterative step, the iterations stop, that is we iterate until convergence. If the initial grouping did not include all biologically relevant specificity groups, some sequences may remain unassigned to any of them (only to the virtual group).

Given a MSA without any additional information, SDPtree randomly selects several groups of equal size, whose number is roughly proportional to the number of sequences in the MSA, and runs SDPgroup. This step is repeated many times (by default, 10000). The distance between two sequences is defined as the negative logarithm of the frequency of assigning them to the same group by the SDPgroup procedure:

(9) $$\begin{array}{l} d(seq_{1},seq_{2})=\\ =-\log\left(\frac{\#\left(seq_{1}\text{ and }seq_{2}\text{ are in the same specificity group}\right)}{10000}\right). \end{array}$$

Using thus defined distance, we construct a tree using a standard UPGMA procedure. Then we perform tree-guided clustering, so that clusters comprise branches of the tree. We then select the lexicographically best clustering using the following original algorithm.

Let *N *be the set of all sequences (points to be clustered). For every *i*, *j *∈ *N*, the distance *d*_*ij *_is defined by (9). Let *X*, *Y *⊆ *N *be subsets of *N*. We define the distance between *X *and *Y *by *d*(*X*, *Y*) = *min *{*d*_*ij*_: *i*∈*X*, *j*∈*Y*}. Then for any cluster *X *we can introduce its quality function *Q(X) *as *Q*(*X*) = *Q*_ext _- *Q*_int_, where *Q*_ext _= *d*(*X*, *N *- *X*) and *Q*_int _= *max*{*d*(*Z*, *X *- *Z*):*Z *⊂ *X*}= *diam*(*X*). Thus, effectively,

(10) $$\begin{array}{l} Q(X)=\\ =min\{d_{ij}:i\in X,J\notin X\}-max\{d_{ij}:i,j\in X\}>0. \end{array}$$

The goal function of the clustering procedure is $\{Q(N_{1}),Q(N_{2}),\ldots ,Q(N_{k})\}\underset{lex}{\to }max$, where *N*_1_, *N*_2_,...,*N*_*k *_is a set of non-overlapping subsets of *N*, the union of which constitutes the whole *N*. We compare these sets lexicographically.

For each subtree of the cluster tree, we can define its quality function *Q(X) *as above. Starting from leaves of the tree, we can identify the set of clusters of the maximal quality by the following dynamic programming procedure. We define

(11) $$\begin{array}{l} Q_{\max}(X)=\\ =max\{Q(X),min\{Q_{max}(X_{1\text{eft}}),Q_{max}(X_{\text{right}})\}\}, \end{array}$$

where *X*_left _and *X*_right _are the subtrees corresponding to the left and right children of the node *X*. When we reach the root, we know the maximal weight for each path. Then, backtracking from the root to the leaves, we identify the corresponding clusters by picking the first node *X*, for which *Q(X) *= *Q*_*max *_(*X*). It can be shown that for any quality function *Q*(*X*) and any cluster tree this procedure yields the best (lexicographically maximal) clustering that conforms the cluster tree. This holds for any *Q(X) *that depends only on *X *and not on the clustering of the rest *N *- *X*.

SDPclust is implemented as a Web-server http://bioinf.fbb.msu.ru/SDPfoxWeb/ and a stand-alone Java application that can be downloaded from the same site. The Web interface is designed for an easier problem of the training set-guided specificity prediction, as described in the SDPgroup procedure. The alignment may contain from 4 to 2000 sequences forming at most 200 specificity groups, and must be shorter that 5000 aa. The more time-consuming *ab initio *grouping is implemented in the stand-alone application SDPfox.jar. It requires Java version 1.6.0_17 installed. To get help, run java -jar SDPfox.jar.

### Benchmark datasets

#### Generated data

**Figure 2:**
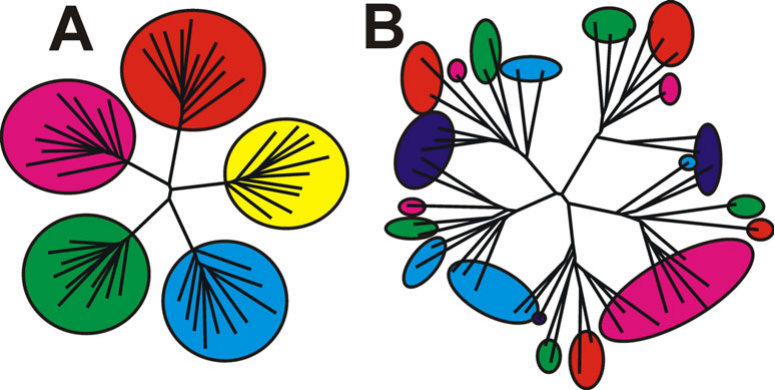
**Phylogenetic trees for artificially generated families, where specificity correlates with subfamilies (A), or is randomly distributed among them (B)**. Same colors indicate coinciding specificity.

We generated two families of sequences of 190 amino acid in length following a naïve random evolutionary model. A random sequence of 190 amino acids was generated using amino acid frequencies from SwissProt. Then on each step the amino acid at a random position was mutated to a random amino acid 30 times. The resulting sequences were used as seed for a subsequent mutation process of another 50 steps. Thus, each family contained five subfamilies of ten sequences each differing from each other in up to 80 positions. Specificity was implicitly introduced by adding ten SDPs to each sequence to follow a pre-defined phylogenetic pattern: in one case specificity coincided with the subfamilies, in the other it was randomly distributed among them (Figure 2, Additional Files 1 and 2).

#### LacI family

Transcription factors of the LacI family regulate sugar catabolism and several other metabolic pathways in a wide variety of bacterial species. They bind DNA operator sequences responding to the effector molecule. We considered a subset of proteins, differing in their specificity to the effector and the operator sequence, that included ten ortholog rows CcpA, CytR, FruR, GalR, GntR, MalR, PurR, RbsR_EC, RbsR_PP, ScrR (Additional File 3). The RbsR_EC and RbsR_PP groups represent two groups of ribose repressors from different bacterial lineages (Enterobacteriales, Vibrionales and Pasteurellales, and Pseudomonadales, respectively) that share the ligand specificity but have different DNA binding motifs, thus being considered as separate groups in our study.

### Enzyme datasets

To benchmark SDPclust against other methods we used two datasets of 18 (Dataset 1) and 26 (Dataset 2) Pfam seed alignments. Dataset 1 contains Pfam families, in which all proteins have the same EC number, and Dataset 2 consists of the families that include enzymes from at least two EC categories differing in the last term. Additionally, we required that for each of these families, the 3 D structure of one of its members with bound ligand were available. These families are listed in Additional File 4 and described in detail elsewhere [18]. This dataset is different from the enzyme dataset described in [19] in that all the structures include a bound native ligand. So we believe it is more suitable for benchmarking a method for prediction of specificity determinants.

### Benchmark criteria

After applying SDPclust, one obtains two resulting predictions: the SDP set and the specificity groups. We apply the standard sensitivity and false positive measures to assess the prediction of SDPs.

The sensitivity is given by the formula TP/(TP + FN), and the false positive rate by FP/(FP + TN), where TP is the number of true positives (i.e. residues both belonging to the gold standard positive set and predicted as positives), FP is the number of false positives, TN is the number of true negatives and FN is the number of false negatives. To evaluate the predicted grouping, we use the MI-based metric given by the formula

(12) D(Greal,Gpred)==1−MI(Greal,Gpred)/H(Greal,Gpred),

where *G*_real _is the gold standard grouping, *G*_pred _is the predicted grouping, and H and MI are the joint Shannon entropy and mutual information of the two distributions, respectively [20]:

(13) H(Greal,Gpred)=−∑ijpijlogpij,MI(Greal,Gpred)==H(Greal)+H(Gpred)-H(Greal, Gpred)==∑i,jpijlogpijpipj,

where *p*_*ij *_denotes the probability of a sequence to be in the i-th group of *G*_real _and j-th group of *G*_pred_. Here we treat groupings as random variables over the space of sequences in the MSA. Defined so, this distance is a metric, ranged from 0 (for identical distributions) to 1, as shown in [20], and reflects proximity of two distributions, in our case, the gold standard and the predicted grouping of sequences.

In the case of generated families we have the standard of truth given artificially: the ten introduced SDPs, and the induced grouping.

For the LacI family, we used the results of extensive mutational analysis of LacI from *E. coli *[21] and defined true SDPs as all positions, whose mutation resulted in a weakening the function of the protein (groups IV to XV from [21]). We are aware of the fact that many functionally important positions probably account for the functionality that is common for all LacI proteins, and thus are likely to be conserved in the family. So, many positions that are defined here as 'positives' are in fact 'negatives', which leads to underestimation of the results. However, biochemical data on real specificity determinants that would cover a protein to a significant part of its length is largely non-existent, so we decided to allow for this shortcoming in the definitions, despite the fact that it may make the performance to appear worse than it might be.

The gold standard grouping was derived from the comparative genomics studies [22]. Briefly, a transcription factor was assumed to be specific to a certain effector, if its gene was found in the same locus as genes of the corresponding pathway, or if it shared a DNA regulatory motif with these genes.

As such functional data are not available for the enzyme datasets, we define true SDP as all residues that are located closer than 10Å to the ligand in the corresponding 3 D structure. This likely underestimates the method's sensitivity. We assess the quality of the grouping in the enzyme dataset using formulae (8)-(9) and considering only sequences with a known EC number as a subject for this assessment.

### Application

As a real-world example to test our approach, we selected a family of phosphodiesterases (PDEs). PDEs catalyze hydrolysis of cAMP and/or cGMP and are implicated in various diseases. PDEs comprise at least eleven subfamilies with different and partially overlapping specificity to the cyclic nucleotides. The human genome contains 21 genes encoding PDEs [23]. The sequences were selected from the Pfam entry PF00233 so that no two sequences were more than 95% identical, and all sequences were long enough to cover at least 70% of the alignment. The resulting alignment contained 249 sequences, 42 of which were annotated as belonging to a certain PDE subfamily in Swissprot. The alignment is presented in Additional File 5.

When analyzing contact in structures of LacI and PDE family members, we always assume 5Å to be the cutoff for two atoms to be contacting each other, and contacting residues are defined by the contact of their nearest atoms.

## Results

### Performance of SDPclust in benchmark cases

#### Generated data and the LacI family

The sensitivity and false positive rate of the set of predicted SDPs and the MI-based distance between the gold standard and the predicted groupings are given in Table 1. SDPclust performs correctly for both generated families, but demonstrates a lower sensitivity for the real-world example of the LacI family. This may be caused by our definition of true SDPs, as all residues, whose mutation resulted in alteration of function [21].

**Table 1:** Benchmark results for the artificially generated data and the LacI family.

	Sensitivity	False positive rate	Distance
Generated family 1	1.00	0.00	0.00
Generated family 2	1.00	0.00	0.00
LacI family	0.15	0.03	0.08

For the generated data, SDPs were introduced after the mutation process, for the LacI family, all positions that alter protein function according to [21] were considered SDPs.

**Figure 3:**
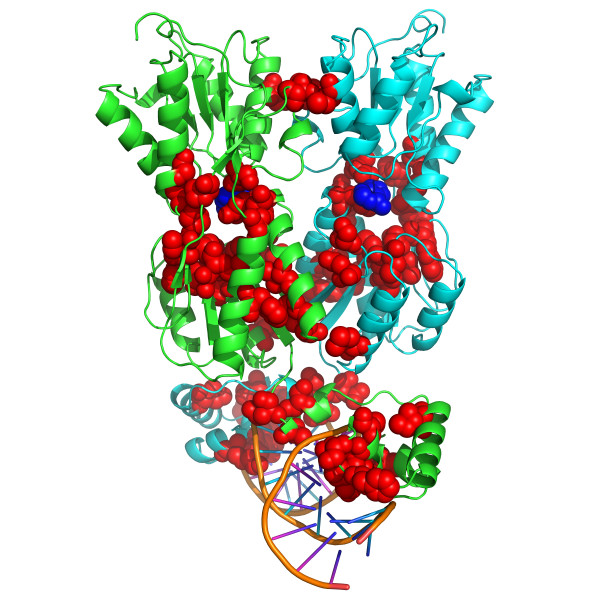
**Mapping of the predicted SDPs on the structure of PurR from *E. coli***. Two subunits of the dimer are colored green and cyan. DNA is shown in orange. SDPs are shown in red. Obviously, not all of them confer specificity, which results in considerable underestimation of sensitivity.

Additionally, we mapped the predicted SDPs for the LacI family onto the 3 D structure of PurR from *E. coli *(PDB ID 1BDH, Figure 3). As expected, the predicted SDPs were located in two functionally important regions of the protein: in the DNA-binding domain and in the effector-binding pocket. Seven and two amino acid residues directly contacted DNA and effector, respectively. Additional four residues were involved in intersubunit contacts.

### Enzyme datasets

The average values of sensitivity and false positive rate, and the average and median distance to the ligand are given in Table 2. The statistical significance of the prediction was also calculated applying the Mann-Whitney test to the distances from the ligand to the predicted SDPs and to all residues from the structure. For 21 out of 44 considered families, the p-values were lower than 0.1, and for 10 families they were below 0.01. This indicates that SDPclust performs significantly better than random choice. Dataset 2, which corresponds to families that include proteins with different specificity, is enriched in the statistically significant predictions: 15 out of 21 with p-value below 0.1, and 7 out of 10 with p-value below 0.01 come from this set. Taken together, in cases, when there is indication of different specificities within a family, SDPclust proves to be a powerful tool for exploring both specificity groups and SDPs.

**Table 2:** Average benchmark results for the enzyme datasets.

	Sensitivity	False positive rate	Average distance to the ligand	Median distance to the ligand
Dataset 1	0.11	0.07	13.77	12.5
Dataset 2	0.11	0.08	12.69	11.87

All positions that lie closer than 10 Å to the co-crystallized ligand in the corresponding structure are considered SDPs.

### Prediction of the protein specificity in the PDE family

Phosphodiesterases (PDEs) catalyze hydrolysis of cAMP and/or cGMP, secondary messengers that regulate a variety of cellular processes, including response to hormones, neurotransmitters, cytokines, chemokines. This makes PDEs an attractive drug target. The human genome encodes 21 PDEs, which differ in their specificity both to the cyclic mononucleotides and to designed inhibitors. We applied SDPclust to predict the amino acid residues accounting for these differences and the specificity of unannotated members of the family.

SDPclust splits the PDE family into 37 specificity groups. PDE subfamilies PDE1, PDE3, PDE4, PDE6, PDE7, PDE8, PDE9, PDE10 form separate specificity groups (that may include some unannotated sequences thus providing potential annotation for them), and subfamilies PDE2, PDE5, PDE11 form two specificity groups each. 23 specificity groups do not contain any annotated sequences. The resulting grouping is available in Additional File 5.

**Figure 4:**
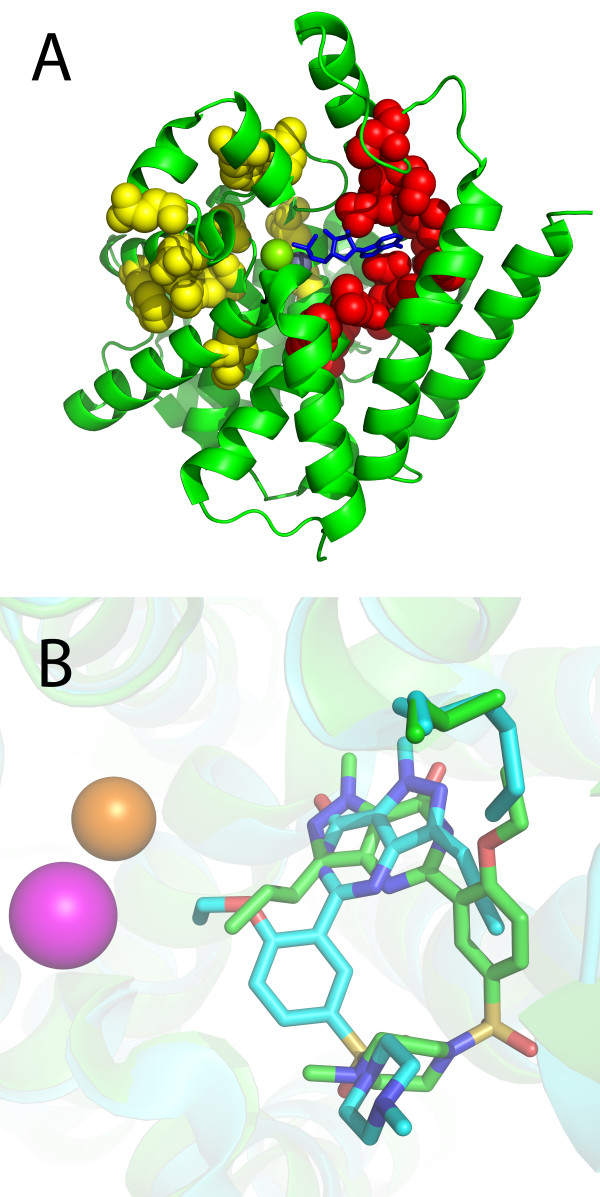
**(A) Mapping of the predicted SDPs onto the catalytic domain of PDE4 D (PDB ID **1TFB**)**. AMP colored blue, SDPs shown as spheres, SDPs located in the hydrophobic ligand-binding pocket colored red, SDPs located on the other side of bimetallic cluster colored yellow. (B) Superposition of sildenafil molecules in active sites of PDE4B (cyan) and PDE5A (green). Sildenafil bound to PDE4B is colored blue, and bound to PDE5A is dark green.

SDPclust identifies 23 SDPs: 615W, 619F, 624C, 652 S, 655L, 658R, 660V, 665I, 677C, 683 H, 686F, 690L, 719A, 761T, 765L, 767A, 768I, 770K, 775Q, 779A, 782V, 783A, 805 M (numbered according to PDE5A from human). Prior to this study, among these SDPs, 775Q, 779A, 782V, 783A, 805 M were experimentally shown to be involved in rolipram resistance in PDE4B [24,25], and 767A and 775Q to be important for cyclic mononucleotide selectivity [26]. Mapped onto the 3 D structure of human PDE4 D (PDB ID 1TB7), SDPs form two spatial clusters: one comprising nine amino acid residues and located in the hydrophobic ligand-binding pocket, and the other comprising 13 residues located on the other side of the bimetallic cluster (Figure 4A). Analyzing 39 3 D structures of different PDEs, we found that 11 SDPs contact the ligand in at least one of them, while only one (782V) contacts it in all considered structures.

PDE4B and PDE5A have different specificity towards the cyclic nucleotide, however, both bind sildenafil (PDB ID 1TBF for PDE4B and PDB ID 1XOS for PDE5A), although PDE5A binds it with much higher affinity [27]. In these two structures, the substrate is bound in two different conformations. Interestingly, one of the predicted SDPs, 783A, can cause steric obstructions, preventing binding of sildenafil to PDE4B in the same conformations as to PDE5A ([27], Figure 4B).

## Discussion

We presented a novel method, SDPclust, for the prediction of protein specificity in large and potentially diverse families. Essentially, the resulting prediction consists of two parts: a set of potential groups that contain proteins with the same specificity (specificity groups), and a set of positions that account for differences in specificity among the proteins (SDPs). We compared the performance of SDPclust to other existing approaches using several benchmark datasets.

The first part of the prediction, the specificity groups, was compared to predictions by SDPsite [18], bete [28], giant component [29], FASS and S-method [12], Protein Keys [17]. We were unable to include the methods by Marttinen and co-workers [15], as there are no functional executables currently available for this method. In all cases, except the giant component analysis (where we had to implement the method in house due to its unavailability online), the executables provided by the authors with the default parameters were used to perform the prediction. We have the gold standard grouping for the artificially generated set and for the LacI family. The resulting MI-based distances are presented in Table 3A. SDPclust performs best in terms of selection of specificity groups, including the difficult case of the generated family 2, where other methods fail. The reason for this might be that SDPclust does not assume that specificity should by monophyletic. This allows for successful resolution in difficult cases of convergent evolution of specificity or uncertain branching in the phylogenetic tree.

**Table 3:** MI-based error of subfamily-identifying methods.

A. Generated data and LacI								
	**SDPsite**	**bete**	**Giant component**	**Protein keys**	**FASS**	**S-method**	**SDPclust**	

Generated family 1	0.58	0.00	1.00	0.00	0.00	0.00	**0.00**	
Generated family 2	0.94	1.00	1.00	0.94	0.95	0.94	**0.00**	
LacI	0.11	0.12	0.20	0.16	0.93	1.00	**0.10**	

**B. Enzyme dataset**								

	**SDPsite**	**Giant component**	**Protein keys**	**FASS**	**S-method**	**SDPclust**	**# EC**	**# sequences**

PF00108	**0.000**	0.633	0.621	0.687	0.786	0.679	2	22

PF00128	0.801	0.552	0.586	N/A	N/A	**0.544**	10	154

PF00135	**0.429**	0.583	0.548	N/A	N/A	0.486	4	129

PF00215	0.759	0.878	0.803	N/A	0.758	**0.751**	3	92

PF00278	0.321	0.608	0.538	0.311	0.577	**0.277**	3	55

PF00293	0.239	0.449	0.200	N/A	N/A	**0.237**	6	205

PF00348	1.000	**0.352**	0.555	0.674	0.764	0.372	3	16

PF00351	**0.292**	1.000	0.495	N/A	N/A	0.573	3	6

PF00579	0.492	0.749	0.603	0.629	0.764	**0.472**	2	41

PF00583	**0.132**	0.326	0.261	N/A	N/A	0.311	10	244

PF00590	1.000	0.383	0.141	0.603	0.561	**0.070**	7	22

PF00755	0.544	**0.407**	0.522	N/A	N/A	0.431	4	22

PF00871	1.000	**0.675**	0.709	N/A	N/A	0.752	3	12

PF00896	**0.000**	0.594	**0.000**	N/A	N/A	**0.000**	2	13

PF00962	**0.000**	0.654	0.399	0.285	0.367	0.494	2	17

PF01048	**0.000**	0.722	0.571	0.912	0.912	0.912	2	16

PF01112	**0.000**	0.500	0.333	N/A	N/A	0.333	2	7

PF01467	0.756	0.515	0.432	0.543	0.295	**0.142**	6	67

PF01712	0.500	**0.387**	0.521	N/A	N/A	0.500	4	14

PF02274	**0.000**	0.788	0.707	0.000	1.000	0.707	2	32

PF03171	0.668	0.096	0.196	0.812	0.813	**0.075**	11	153

Overall distance*0.184	0.276	0.204	0.250	0.172	**0.165**			

The following Web-servers were used for testing: SDPsite http://bioinf.fbb.msu.ru/SDPsite/index.jsp, bete http://phylogenomics.berkeley.edu/cgi-bin/SCI-PHY/input_SCI-PHY.py, giant component (unavialable, implemented in house), Protein Keys http://www.proteinkeys.org/proteinkeys/, FASS, S-method http://treedet.bioinfo.cnio.es/, SDPclust http://bioinf.fbb.msu.ru/SDPfoxWeb/main.jsp. The best performing method is marked in bold.

* all groups are treated together, as members of the same family.

We compared different methods that predict specificity groups for the enzyme dataset 2 (different EC numbers in the same family) (Table 3B). We considered only families with at least four sequences with assigned EC, which left us with 21 families. One can see that SDPclust and SDPsite perform comparably well. There is one clear tendency in the SDPclust performance: all families, for which SDPclust performs significantly worse than SDPsite, contain 2 specificity groups (as defined by EC numbers) and a relatively small numver of sequences (<25). Generally, SDPclust tends to perform better on larger families, due to the fact that it favors splits to many specificity groups. Very rarely it puts sequences with different EC numbers together, but can put sequences with the same EC number into different clusters. On the contrary, SDPsite performs best when there are only two groups and few sequences. This suggests possible complementarity of these methods.

**Figure 5:**
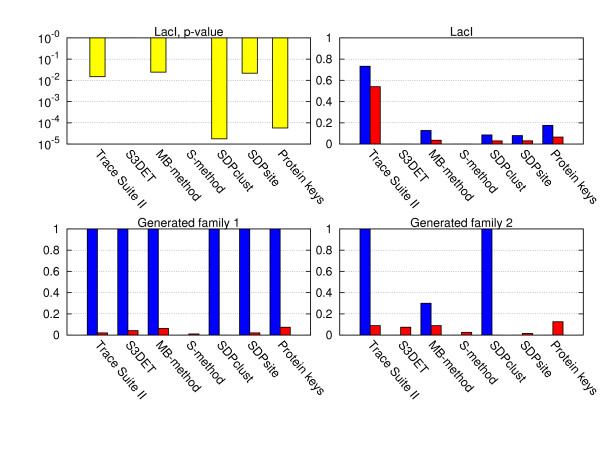
**Sensitivity, false positive rate and statistical significance for the artificially generated families and the LacI family**. Yellow denote p-value, blue, sensitivity and red, false positive rate. The statistical significance can be computed only for the LacI family, since it involves calculating distance to the ligand bound in the 3 D structure. FASS and S-method predict zero residues for the LacI family.

The predicted SDPs were compared to functionally important positions identified by SDPsite [18], FASS, MB-method and S-method [12], Trace Suite II (implementing the method described in [2]), Protein Keys [17]. Each of these methods produces a set of residues that are potentially important for functional differences between subfamilies of a given alignment. None requires prior grouping into subfamilies. We also computed statistical significance of the derived predictions for the LacI family applying the Mann-Whitney test to distances of the predicted SDPs to the functional site of the protein compared to all amino acid resudues of the regulator. The sensitivity and false positive rate values and the statistical significance for the artificially generated data and the LacI family are presented in Figure 5. For generated family 1, the sensitivity of all methods (except S-method) is 1, whereas only SDPclust has a false positive rate of 0. In contrast to this, for generated family 2, only Trace Suite II and SDPclust have the sensitivity of 1, and MB-method, of 0.3, while the remaining methods fail completely (probably, due to their subfamily extraction procedures). Again, SDPclust is the only method to show the false positive rate of 0. For the LacI family, Trace Suite II has the highest sensitivity (0.73), but also a very high false positive rate (0.54), which makes it impractical to use. SDPsite, MB-method and SDPclust have comparable sensitivities and false positive rates, but SDPclust is the only method, whose predictions are significant according to our statistical significance test that assesses proximity to the ligand (p-value = 8 × 10^-5^). It must be also noted that the FASS, MB and S-methods turned out to be inapplicable to many instances from out benchmark dataset, due to restrictions imposed onto the alignment length and the number of sequences, while demonstrating good performance in the remaining cases.

**Figure 6:**
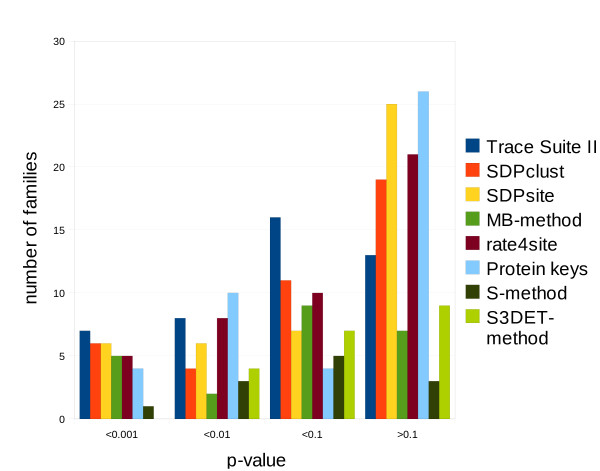
**Statistical significance of predictions by different methods for benchmark datasets 1 and 2**.

The average sensitivity, false positive rate and statistical significance of different methods for the combined enzyme dataset are presented in Table 4, and the complete statistics is given in Additional File 6. SDPclust shows a relatively high sensitivity and a relatively low false discovery rate for both datasets. A detailed analysis of the statistical significance is presented in Figure 6. The histogram shows the fraction of families from the enzyme dataset, for which the prediction of different methods have p-values below a certain point. Trace Suite II demonstrates low p-values, but, again, a high false positive rate. In other words, this method predicts so many positions (part of which are functionally important) that it barely narrows the set of potentially important residues, which makes it impractical in real-world studies. FASS, MB and S methods are inapplicable to a large fraction of families, which makes comparison to them uninformative. Rate4site, SDPsite, Protein Keys and SDPclust perform comparably well, SDPclust being slightly better in the case of the multi-EC dataset. This can be due to the refined grouping procedure. Noteworthy, SDPsite and SDPclust are complementary to rate4site, which predicts functionally important positions on the basis of their conservation, in the sense that they identify another set of functionally important residues, the ones accounting for differences in specificity. This is supported by the observation that rate4site performs better for dataset 1 (same specificity for all family members), whereas SDPclust and SDPsite perform better for dataset 2 (at least two different specificities within the family) (data not shown).

**Table 4:** Sensitivity, false positive rate, average and median distances to the ligand for predictions obtained with different methods for the enzyme dataset.

	Sensitivity	False positive rate	Average distance to the ligand	Median distance to the ligand
dataset1				

Trace Suite II	0.78	0.55	12.27	11.77

FASS	0.04	0.01	9.82	11.42

MB-method	0.13	0.05	9.67	8.67

S-method	0.04	0.01	9.66	12.92

rate4site	0.26	0.16	11.91	11.74

SDPclust	0.10	0.04	10.96	10.96

SDPsite	0.14	0.13	12.71	12.62

dataset2				

Trace Suite II	0.69	0.51	12.26	11.88

FASS	0.07	0.01	8.80	9.62

MB-method	0.15	0.06	9.89	9.86

S-method	0.07	0.01	6.84	6.90

rate4site	0.21	0.15	12.59	12.40

SDPclust	0.12	0.06	10.81	11.08

SDPsite	0.16	0.11	11.46	11.17

All positions that lie closer than 10 Å to the co-crystallized ligand in the corresponding structure are considered SDPs.

## Competing interests

The authors declare that they have no competing interests.

## Authors' contributions

PVM participated in the design of the algorithms and data analysis, performed the programming and drafted the manuscript. MSG participated in the data collection and analysis and manuscript preparation. AAM and ARR participated in the design of the algorithms. ABR and RBR participated in the data collection. OVK participated in the design of the algorithms, data collection, and analysis and preparation of the manuscript. All authors have read and approved the final manuscript.

## Supplementary Material

Additional file 1 **Sequence alignment for generated sequences, specificity coincides with phylogeny**. Click here for file

Additional file 2 **Sequence alignment for generated sequences, specificity does not correlate with phylogeny**. Click here for file

Additional file 3 **Sequence alignment for the LacI family divided into specificity groups**. Click here for file

Additional file 4 **Families of the benchmark datasets**. Click here for file

Additional file 5 **Sequence alignment for the PDE family divided into predicted specificity groups**. Click here for file

Additional file 6 **Detailed results for the benchmark datasets**. Click here for file
