# Supplementary material for: An automated stochastic approach to the identification of the protein specificity determinants and functional subfamilies
Source: Algorithms Mol Biol. 2010 Jul 15;5:29. doi: 10.1186/1748-7188-5-29 (PMC2914642; doi:10.1186/1748-7188-5-29)
Supplement: Additional file 4 — Families of the benchmark datasets. [file 1748-7188-5-29-S4.PDF]

a. Diverse dataset, two or more EC numbers per family

Family ID	Family name	# sequences	Alignment length	ECs	PDB	Bound ligand equivalent to natural substrate/ product
PF00108	Thiolase_N	22	291	2.3.1.9<br>2.3.1.16<br>2.3.1.176	1NL7	Coenzyme A
PF00128	Alpha-amylase	54	673	2.4.1.4<br>2.4.1.7<br>3.2.1.10<br>3.2.1.20<br>3.2.1.70<br>3.2.1.98<br>3.2.1.93<br>3.2.1.141<br>5.4.99.16<br>5.4.99.15	2D3N	Glucose
PF00135	COesterase	129	889	3.1.1.1<br>3.1.1.3<br>3.1.1.7<br>3.1.1.8<br>3.1.1.13<br>3.1.1.59	1P0M	Choline ion
PF00215	OMPdecase	92	402	4.1.1.23<br>4.1.1.85	2CZE	Uridine-5'-monophosphate
PF00278	Orn_DAP_Arg_deC	55	220	4.1.1.17<br>4.1.1.18<br>4.1.1.19<br>4.1.1.20	1TWI	Lysine
PF00293	NUDIX	205	314	2.7.7.1<br>3.6.1.13<br>3.6.1.17<br>3.6.1.52<br>3.6.1.52<br>5.3.3.2	2DSC	Adenosine-5-diphosphoribose
PF00348	polyprenyl_synt	16	289	2.5.1.10<br>2.5.1.29	2F8Z	Zoledronic acid, 3-methylbut-3-enyl trihydrogen diphosphate
PF00351	Biopterin_H	6	332	1.14.16.1<br>1.14.16.2<br>1.14.16.4	1MMK	5,6,7,8-tetrahydrobiopterin, beta(2-thienyl)alanine
PF00579	tRNA-synt_1b	41	402	6.1.1.1<br>6.1.1.2	1WQ4	Tyrosine
PF00583	Acetyltransf_1	244	150	2.3.1.1<br>2.3.1.4<br>2.3.1.48<br>2.3.1.57<br>2.3.1.59<br>2.3.1.82<br>2.3.1.87<br>2.3.1.88	1TIQ	Coenzyme A

				2.3.1.128		
PF00590	TP_methylase	22	247	2.1.1.98<br>2.1.1.107<br>2.1.1.130<br>2.1.1.131<br>2.1.1.132<br>2.1.1.133<br>2.1.1.152<br>2.1.1.151<br>4.2.1.75<br>4.99.1.4	1S4D	S-adenosyl-L-homocysteine
PF00755	Carn_acyltransf	22	867	2.3.1.6<br>2.3.1.7<br>2.3.1.21<br>2.3.1.137	1NDI	Coenzyme A
PF00871	Acetate_kinase	12	405	2.7.2.1<br>2.7.2.7<br>2.7.2.15	1TUY	Adenosine-5'-diphosphate
PF00896	Mtap_PNP	13	288	2.4.2.1<br>2.4.2.28	1V48	9-(5,5-difluoro-5-phosphonopentyl)guanine
PF00962	A_deaminase	17	475	3.5.4.4<br>3.5.4.6	1NDZ	1-((1r)-1-(hydroxymethyl)-3-(6-((3-(1-methyl- 1h-benzimidazol-2-yl)propanoyl)amino)-1h- indol-1-yl)propyl)-1h-imidazole-4-carboxamide
PF01048	PNP_UDP_1	16	276	2.4.2.1<br>2.4.2.3<br>2.4.2.28<br>3.2.2.4<br>3.2.2.9	1PK7	Adenosine
PF01112	Asparaginase_2	7	365	3.5.1.1<br>3.5.1.26	1SEO	Aspartic acid
PF01135	PCMT	9	232	2.1.1.77<br>2.1.1.36	1R18	S-adenosyl-L-homocysteine
PF01202	SKI	100	263	2.7.4.3<br>2.7.1.12<br>2.7.4.14<br>2.7.1.71<br>4.2.3.4	1WE2	Adenosine-5'-diphosphate
PF01234	NNMT_PNMT_TEMT	7	289	2.1.1.1<br>2.1.1.28<br>2.1.1.49	2AN4	S-adenosyl-L-homocysteine
PF01467	CTP_transf_2	66	302	2.7.7.1<br>2.7.7.3<br>2.7.7.14<br>2.7.7.15<br>2.7.7.18<br>2.7.7.39	1N1D	[Cytidine-5'-phosphate] glycerylphosphoric acid ester
PF01712	dNK	14	174	1.6.99.3<br>2.7.1.21<br>2.7.1.74<br>2.7.1.76<br>2.7.1.113<br>2.7.1.145	2A2Z	Uridine-5'-diphosphate, 2'-deoxycytidine

PF02274	Amidinotransf	32	455	2.1.4.1<br>3.5.3.6<br>3.5.3.18	2A9G	Arginine
PF03061	4HBT	153	102	3.1.2.2<br>3.1.2.23	1LO7	2-oxyglutaric acid, 2-aminoethanesulfonic acid
PF03171	2OG-FeII_Oxy	147	183	1.14.11.2<br>1.14.11.4<br>1.14.11.7<br>1.14.11.9<br>1.14.11.11<br>1.14.11.13<br>1.14.11.19<br>1.14.11.20<br>1.14.11.23<br>1.14.11.26<br>1.14.17.4<br>1.14.20.1<br>1.21.3.1	2FDJ	4-hydroxyphenacyl coenzyme A
PF03414	Glyco_transf_6	6	341	2.4.1.87<br>2.4.1.40	1LZJ	Succinic acid

b. Homogeneous dataset, strictly one EC number per family

Family ID	Family name	# sequences	lengthAlignment	EC	PDB	Bound ligand equivalent to natural substrate/ product
PF00303	Thymidylat_synt	19	384	2.1.1.45	2G8O	2'-deoxyuridine 5'-monophosphate, 10-propargyl-5,8-dideazafolic acid
PF00693	Herpes_TK	15	305	2.7.1.21	1VTK	Adenosine-5'-diphosphate, thymidine-5'-phosphate
PF00925	GTP_cyclohydro2	16	193	3.5.4.25	2BZ0	Phosphomethylphosphonic acid guanylate ester
PF01014	Uricase	17	196	1.7.3.3	2FXL	1-(2,5-dioxo-2,5-dihydro-1h-imidazol-4-yl)urea
PF01227	GTP_cyclohydroI	16	107	3.5.4.16	1A8R	Guanosine-5'-triphosphate
PF01293	PEPCK_ATP	12	495	4.1.1.49	1YTM	Adenosine-5'-triphosphate, oxalic acid
PF01583	APS_kinase	20	166	2.7.1.25	1M7G	Adenosine-5'-phosphosulfate, adenosine-5'-diphosphate-2',3'-vanadate
PF01656	CbiA	80	372	6.3.3.3	1A82	Adenosine-5'-triphosphate, 7,8-diamino-nonanoic acid
PF01702	TGT	13	256	2.4.2.29	1Q2S	9-deazaguanine
PF01747	ATP-sulfurylase	19	397	2.7.7.4	1G8H	Adenosine-5'-phosphosulfate, pyrophosphate 2-
PF02110	HK	9	282	2.7.1.50	1ESQ	Adenosine-5'-triphosphate, 4-methyl-5-hydroxyethylthiazole

						phosphate
PF02223	Thymidylate_kin	26	209	2.7.4.9	1E9E	Adenosine-5'-diphosphate, thymidine-5'-phosphate
PF02277	DBI_PRT	28	398	2.4.2.21	1L5L	7-alpha-d-ribofuranosyl-purine-5'-phosphate, nicotinic acid
PF02353	CMAS	12	304	2.1.1.79	1KPI	S-adenosyl-Ll-homocysteine
PF02569	Pantoate_ligase	7	311	6.3.2.1	2A86	Adenosine monophosphate, beta-alanine
PF02898	NO_synthase	8	374	1.14.13.39	1Q2O	L-n(omega)-nitroarginine-2,4-L-diaminobutyric amide
PF02901	PFL	11	734	2.3.1.54	1MZO	Pyruvic acid
PF03332	PMM	9	248	5.4.2.8	2FUE	Alpha-d-mannose 1-phosphate
